# Cannabidiol Treatment for Refractory Epilepsies in Pediatrics

**DOI:** 10.3389/fphar.2020.586110

**Published:** 2020-09-29

**Authors:** Umberto Raucci, Nicola Pietrafusa, Maria Chiara Paolino, Giovanni Di Nardo, Maria Pia Villa, Piero Pavone, Gianluca Terrin, Nicola Specchio, Pasquale Striano, Pasquale Parisi

**Affiliations:** ^1^ Pediatric Emergency Department, Bambino Gesù Children’s Hospital, Istituto di Ricerca e Cura a Carattere Scientifico, Rome, Italy; ^2^ Rare and Complex Epilepsy Unit, Department of Neuroscience and Neurorehabilitation, Member of European Reference Network EpiCare, Bambino Gesù Children’s Hospital, Istituto di Ricerca e Cura a Carattere Scientifico, Rome, Italy; ^3^ Child Neurology, Chair of Pediatrics, NESMOS Department, Faculty of Medicine and Psychology, Sapienza University, Rome, Italy; ^4^ Department of Clinical and Experimental Medicine, Section of Paediatrics & Child Neuropsychiatry, Catania University, Catania, Italy; ^5^ Department of Gynecological Obstetric and Urological Sciences, Faculty of Medicine and Dentistry, Sapienza University of Rome, Rome, Italy; ^6^ Department of Neurosciences, Rehabilitation, Ophthalmology, Genetics, Maternal and Child Health, University of Genoa, ‘G. Gaslini’ Institute, Genova, Italy

**Keywords:** CBD—cannabidiol, drug-drug interaction, drug-resistant epilepsy (DRE), children, epileptic encephalopathy, DRAVET syndrome, Lennox-Gastaut syndrome (LGS)

## Abstract

Cannabis extracts in oil are becoming increasingly available, and, during the last years, there has been growing public and scientific interest about therapeutic properties of these compounds for the treatment of several neurologic diseases, not just epilepsy. The discovered role of the endocannabinoid system in epileptogenesis has provided the basis to investigate the pharmacological use of exogenously produced cannabinoids, to treat epilepsy. Although, physicians show reluctance to recommend Cannabis extracts given the lack of high-quality safety available data, from literature data cannabidiol (CBD) results to be a promising and safe anticonvulsant drug with low side-effect. In particular, according to early studies, CBD can reduce the frequency of seizures and lead to improvements in quality of life in children affected by refractory epilepsy. So, for these reasons, the detailed study of the interactions between CBD and anticonvulsant drugs (AEDs) administered simultaneously in polytherapy, is arousing increasing interest, to clarify and to assess the incidence of adverse effects and the relation between dose escalation and quality of life measures. To date, in pediatric age, CBD efficacy and safety is not supported by well-designed trials and strong scientific evidence are not available. These studies are either retrospective or small-scale observational and only during the last years Class I evidence data for a pure form of CBD have been available, as demonstrated in placebo-controlled RCTs for patients affected by Lennox-Gastaut syndrome and Dravet syndrome. It is necessary to investigate CBD safety, pharmacokinetics and interaction with other AEDs alongside performing double-blinded placebo-controlled trials to obtain conclusive data on its efficacy and safety in the most frequent epilepsies in children, not just in the epileptic encephalopathy. This review was aimed to revise the available data to describe the scientific evidence for CBD in Pediatric Epilepsies.

## Introduction

In the last two decades, public and scientific interest on the use of cannabis-derived products for therapeutic purpose in different disease has increased. More than 100 different phytocannabinoid compounds derived from the marijuana plant, *Cannabis sativa*, and *Cannabis indica* which contain up to 500 chemical species (Husni et al., 2014). Literature data have grown (Elliot et al., 2020) and one of the major fields of interest is on the anti-seizure role of the two main components of cannabis, Δ-9-tetrahydrocannabinol (THC) and cannabidiol (CBD), for refractory epilepsy in the pediatric population (Paolino et al., 2016). THC is a psychoactive agent, and its role on seizure control is controversial because of its effect of exacerbating seizure activity; CBD is a non-psychoactive agent whose antiepileptic properties has been demonstrated by both anecdotal and scientific evidence (Friedman and Devinsky, 2015; O’Connell et al., 2017). Up to 30% of children with epilepsy were resistant to standard antiepileptic drugs (Kwan and Brodie, 2000; Kwan et al., 2010) and treatments options for these children are limited.

The discovered role of the endocannabinoid system in epileptogenesis has provided the basis to investigate the pharmacological use of exogenously produced cannabinoids, to treat epilepsy (Cheung et al., 2019; Huntsman et al., 2020).

Several studies, mainly retrospective or small-scale observational, have shown that CBD, both in isolation as a pharmaceutical-grade preparation or as part of a CBD-enriched cannabis herbal extract, is beneficial in decreasing seizure frequency in children with resistant epilepsy.

More studies are needed for physicians to be comfortable authorizing Cannabis-based therapies to children (Lattanzi et al., 2018; Huntsman et al., 2020).

## European (EMA) and Italian Legislation (AIFA), US (FDA)

The growing interest in the therapeutic potential of cannabis-related products is reflected in recent changes in legislation (Arzimanoglou et al., 2020). Laws regarding the use of raw herbal cannabis, cannabis extracts and cannabinoid-based drugs differ between countries (Specchio et al., 2020), and while the use of herbal cannabis for medicinal purposes is now authorized in different countries, cannabis and cannabis extracts have not been approved by the FDA or the European Medicines Agency (EMA).

In the European Union, in contrast to THC, CBD is not controlled and CBD products are approved it not containing more than 0.2% THC (Arzimanoglou et al., 2020).

In Italy from November 2015, can issue licenses for cultivation, production, possession, and use, and herbal cannabis may be prescribed with medical prescription. Italian legislation has recently approved medical use of Cannabis for some conditions such as pain control, chemotherapy- and radiotherapy-induced nausea and vomiting treatment, appetite stimulation in patients affected by cachexia, anorexia, cancer, or AIDS and for other pathologies, such as glaucoma and Tourette’s syndrome (Baratta et al., 2019).

Physicians considering prescribing cannabis-related products should be fully aware of the relevant legislation since the situation can be complex. Guidelines from recognized national professional associations and or governmental bodies can be extremely helpful.

## Historical Review of the Use of Cannabis to Treat Pediatric Epilepsy

Therapeutic properties of cannabis plants have been known from ancient times with documented use for medical purposes in ancient Chinese books in the Middle East and India for at least 4000 years (Russo, 2017).

In 1840 Dr. William Brook O’Shaughnessy described his observation on the use of cannabis in India to treat infantile spasms in a 40-day old infant, and in 1942, he introduced the use of Cannabis Indical in Britain (O’Shaughnessy, 1843). Despite the Marijuana Tax act of 1937 and cannabis prohibition, several researchers and physicians continue the investigation on the medical use of its components. Finally, in 1990, after the discovery of endocannabinoid system and its role in epileptogenesis, and in neuromodulation with attenuation of brain activity, studies on both animal and human use of cannabinoids took place (Marsicano et al., 2003; Wallace et al., 2003; Russo et al., 2005; Englund et al., 2013; Mechoulam and Parker, 2013; Ibeas Bih et al., 2015; Todd and Arnold, 2016).

CBD has been shown to be effective against generalized tonic, clonic, tonic-clonic seizures and on drug-resistant epilepsy models, manifesting behavioral, EEG and neuroprotective effects in both acute and chronic protocols of experimental animal models (Lazarini-Lopes et al., 2020).

### Efficacy in Epilepsy

The bioactive lipid system, their receptor targets, and the metabolic enzymes responsible for the synthesis and degradation of eCB constitute the so-called “endocannabinoid system” (ECS). Many studies have reported alterations of distinct components of the ECS in both animal models of epilepsy and in humans. Furthermore, compounds that act on the ECS have been shown to be effective against epilepsy. In particular, in several cases, the activation of the ECS seems to prevent seizures and reduce mortality, while the pharmacological block of the ECS exerts a proconvulsive action (Verrotti et al., 2016).

First data derived from anecdotal reports that have inspired families to seek CBD-related compounds for the treatment of their children’s drug-resistant epilepsy (Filloux, 2015). The most well-known report is that of Charlotte, a 5-year-old girl in the US who was diagnosed in 2013 with *SCN1A*-confirmed Dravet syndrome who had more than 50 generalized tonic-clonic seizures. Following 3 months of treatment with high-CBD-strain cannabis extract (named “Charlotte’s Web”), her seizures were reported to have reduced by more than 90% (Maa and Figi, 2014). Other anecdotal reports suggesting that CBD may improve seizure control and alertness, mood and sleep have also been documented (Porter and Jacobson, 2013).

Other studies have investigated the effect of oral cannabis extracts using parental reporting. Press et al. (2015) and Tzadok et al. (2016) in two different studies reported similar results, with a 50% seizure reduction in about 30% of patients. In a retrospective study by Porcari et al. (2018) of 108 children with epilepsy in the US, the addition of CBD oil over an average of 6 months resulted in >50% seizure reduction in 29% patients, with 10% becoming seizure-free.

A recent meta-analysis provides evidence for the therapeutic efficacy of high content CBD treatments (Pamplona et al., 2018). Overall, the studies on CBD-enriched oils indicate a 50% reduction in seizures in roughly 30–40% of patients (Gonzalez-Giraldo and Sullivan, 2020). However, it should be emphasized that these are uncontrolled studies with heterogeneous CBD preparations, the CBD content of which varied significantly and should be underlined the need for appropriately controlled studies.

In a Canadian prospective, open-label trial of a CBD/THC cannabis oil in DS, were treated 20 children affected by DS with a cannabis plant extract product, containing 100 mg/mL of CBD and 2 mg/mL of THC. After 20 weeks of therapy, a significant improvement in quality of life, reduction in EEG spike activity, and median motor seizure reduction of 70.6%, with 50% responder rate of 63%, were noticed. Adverse events, common during titration, included somnolence, anorexia, and diarrhea. Abnormalities of liver transaminases and platelets were observed with concomitant valproic acid therapy (McCoy et al., 2018).

## Purified Cannabidiol (Epidiolex/Epidyolex^®^) Efficacy in Epilepsy

In 2018, CBD was approved by the FDA as add-on antiepileptic drug in 2-year-old children with Dravet syndrome and Lennox-Gastaut syndrome. Later, it was approved also by the EMA in 2019. The purified preparation of CBD is available from GW Pharmaceuticals plc, named Epidiolex/Epidyolex. It has been shown to have good effects against a large spectrum of seizures from animal studies (Rosenberg et al., 2017a).

Data from an open-label, multicenter expanded access program in 214 patients with childhood-onset, drug-resistant epilepsy were reported in 2016 by Devinsky et al. (2016). Among them, 33 patients had a diagnosis of DS and 31 patients of LGS. An overall median reduction of motor seizures of 36.5% was reported (49.8% for DS patients), and five patients were free of all motor seizures (of the patients with motor and atonic seizures, and 39% and 56% showed a >50% reduction of seizures, respectively) (Devinsky et al., 2016).

A randomized, double-blind, placebo-controlled study was conducted to evaluate the use of Epidiolex^®^, a pharmaceutical-grade cannabidiol preparation, in Dravet’s syndrome. The author demonstrated its efficacy showing a decrease in convulsive seizures frequency in the cannabidiol arm, with 5% of patients, compared with 0% in the placebo arm becoming seizure-free (p 1/4 0.08). The treatment was overall well-tolerated, but it is important to underline that in the cannabidiol arm, there were more serious adverse events such as elevated hepatic transaminases (Devinsky et al., 2017a).

One trial assessed its efficacy in atonic seizures in Lennox-Gastaut syndrome showed a median reduction of atonic seizures from baseline of 41.9% in participants treated with 20 mg of CBD/kg per day versus 17.2% in the placebo group (Thiele et al., 2018).

The efficacy of CBD in reducing seizures frequencies and in improving the quality of life in childhood epilepsy (QOLCE) scores was showed in a systematic review on 17 observational studies (Stockings et al., 2018). Moreover, four clinical trials in children with Dravet and Lennox-Gastaut syndromes showed a higher rate of seizure frequency reduction in CBD treated patients (Lattanzi et al., 2019). A study from Pietrafusa et al. (2019) on artisanal medical CBD oil in patients with developmental and epileptic encephalopathy (DEE) suggest that CBD may have beneficial effects in patients with DEE and an acceptable safety profile.

## Pharmacodynamic and Pharmacokinetics of Cannabidiol and Drug-Drug Interaction

Although the precise mechanisms responsible for the antiseizure effects of CBD remain unclear, a multimodal mechanism of action of CBD in epilepsy had been proposed. Pharmacological data supporting the role of three targets, namely Transient receptor potential vanilloid-1 (TRPV1), the orphan G protein-coupled receptor-55 (GPR55) and the inhibition of adenosine reuptake. As TRPV1 agonist, CBD lead to a decrease of extracellular calcium influx through a TRPV1 channels desensitization, reducing, consequently, neuronal hyperactivity. CBD reduce neuronal hyperexcitability in epileptic tissue as GPR55 antagonist, inhibiting then intracellular calcium release. Finally, CBD depresses neuronal excitability, reducing adenosine uptake and increasing extracellular adenosine concentration, blocking the equilibrative nucleoside transporter ENT1. Other mechanics of action have been proposed: blockade of voltage-gated sodium channels, interactions with voltage-gated potassium channels, 5-HT1a receptors, and α3 and α1 glycine receptors, blockade of T-type calcium channels, modulation of voltage-dependent anion selective channel protein, and modulation of tumor necrosis factor alpha release (Alcorn et al., 2019; Franco and Perucca, 2019; Gray and Whalley, 2020).

Cannabidiol has a lipophilic structure, a variable absorption rate and extensive empathic first-pass metabolism by isozymes CYP2C19 and CYP3A4, explaining its poor oral bioavailability (Jiang et al., 2013). The pick plasma concentration after oil formula oral administration is at 2.5 hours with a biphasic elimination (initial half-life of 6 hours ant terminal half-life of 18-32 hours) related to its distributive process into different tissues (Devinsky et al., 2014; Devinsky et al., 2018a).

CBD may exhibit numerous interactions with AEDs (Johannessen and Landmark, 2010; Johannessen Landmark and Patsalos, 2010; Johannessen Landmark et al., 2012).

CBD has been found to inhibit at clinically relevant concentrations the activity of CYP2C8, CYP2C9, CYP2C19, and CYP2D6 (Franco and Perucca, 2019).

The most clinically significant interaction between CBD and other concomitantly used drugs, based on clinical trials, is with clobazam. CBD, *via* enzyme inhibition (CYP2C19), may lead to an increase (up to five-fold) in its less potent metabolite, N-desmethylclobazam (Geffrey et al., 2015), leading to toxicity principally manifesting as sedation (Gaston et al., 2017). Also, concurrent clobazam may lead to increased 7 hydroxy-cannabidiol (an active metabolite of CBD) (Morrison et al., 2019), which arguably may lead to better seizure control by boosting the effect of CBD; however, studies with and without clobazam are needed.

There are still some unanswered questions regarding the pharmacology of CBD (Landmark and Brandl, 2020; Lattanzi S. M. et al., 2020b), and the clinical impact of its interactions with other drugs in the individual patient is difficult to predict.

In recent reports, addition of CBD increases the AUC of stiripentol by 55% and the plasma brivaracetam concentrations by 95–280%: This interaction could be related to inhibition of CYP2C19 by CBD (Franco and Perucca, 2019).

Only one retrospective, small cohort study, suggested that CBD may increase the plasma levels of topiramate, rufinamide, zonisamide, and eslicarbazepine (Gaston et al., 2017). The evidence of the effect of CBD on valproic acid are conflicting (Morrison et al., 2019).

Considering the effect of CBD on other type of drugs a possible elevation in plasma warfarin concentration should be considered, probably due to inhibition of CYP2C9 (Damkier et al., 2019).

On the other hand, the rule of enzyme-inducing AEDs (carbamazepine and phenitoine) need to be formally investigated. Stiripentol decreases in the levels of two CBD metabolites, 7-carboxy-CBD and 7-hydroxy-CBD (Morrison et al., 2019).

Finally, a rule of rifampicin as inductor and of ketoconazole as inhibitor had been demonstrated (Stott et al., 2013).

Based on the available studies, the difference in the pharmacokinetics of CBD in developmental age compared to adults is difficult to interpret. The pharmacokinetics of pure GW CBD have been evaluated in children with DS aged 4–11 years, who were randomized to different doses. CBD was administered twice daily in addition to background antiepileptic drugs (AEDs), represented mainly clobazam and valproate. Pharmacokinetic evaluations were based on sparse concentration data obtained on day 22, at the end of the maintenance period. Plasma CBD concentrations increased in an approximately dose-proportional manner across the three investigated dose groups (5, 10, and 20 mg/kg/day). Variability in CBD exposure among subjects was considerable, with coefficient of variation in AUC being in the order of 20–121%. 7-carboxy-CBD was the most abundant metabolite in plasma, with concentrations 13- to 17-fold higher than those of CBD. AUC values for 6-hydroxy-CBD were < 10% those of CBD, and those of 7-hydroxy-CBD were also lower respect CBD (Devinsky et al., 2018a).

CBD is related to some risk. While in animal models, CBD serious adverse events such as developmental toxicity, embryo-fetal mortality, central nervous system inhibition and neurotoxicity, hepatocellular injuries, spermatogenesis reduction, and hypotension have been demonstrated, they have been linked to the use of doses higher than human therapies. Human CBD studies reported only mild CBD adverse effects such as hepatic abnormalities, diarrhea, fatigue, vomiting, and somnolence (Huestis et al., 2019).

Patients should be systematically questioned about efficacy, tolerability, and adherence, and serum concentrations should be measured if possible and dosages adjusted accordingly to optimize each patient’s treatment.

## Drug-Resistant Pediatric Epilepsy

### Dravet Syndrome

FDA and EMA approved CBD use in patients suffering from DS based on the results of a randomized, double-blind, placebo-controlled trial performed on 120 DS subjects aged 2–18 years (GWP-CARE1 part B) (Devinsky et al., 2017b).

Patients were administered 20 mg/kg/day CBD over a 14-week titration plus maintenance period, and data were compared to the baseline period. The dose of 20 mg/kg/d was set by an independent drug safety monitoring committee based on pharmacokinetic and safety data from an initial part of this study (Part A). The median frequency of convulsive seizures per month decreased from 12.4 to 5.9 with CBD (from 14.9 to 14.1) and the 43% of patients with CBD had at least a 50% reduction in convulsive seizure frequency (27% in placebo group). In GWPCARE2 patients matched in three arms: patients received CBD at a dose of 10 mg/Kg/day, patients received 20 mg/Kg/day and patients receiving placebo. For CBD 10 group and CBD 20 group patients obtained respectively the 48.7% and 45.7% percentage reduction from baseline in convulsive seizure frequency. The conclusions were that adjunctive CBD at doses of 10 and 20 mg/kg/day led to similar convulsive seizure frequency; safety and tolerability profile was better in 10-mg/kg/day dose group (GWPCARE2; Devinsky et al., 2019).

Patients who completed GWPCARE1 part A or part B or GWPCARE2 were invited to enroll in a long-term open-label extension trial, GWPCARE5 (GWPCARE5; Devinsky et al., 2019). Data from an interim analysis were published. Two hundred and sixty out of 278 patients (95%) who had completed the original randomized trials were enrolled in the open-label extension. In patients from GWPCARE1 part B, over a 48-week periods, the median reduction in monthly seizure frequency ranged from 38 to 44% for convulsive seizures and 39 to 51% for total seizures. The 84% of patients/caregivers reported improvement in the patient’s overall condition on the subject/caregiver GCI scale. The long-term effect of add-on CBD at up to 25–50 mg/kg/day over 144 weeks was reported for DS and LGS patients (Laux et al., 2019). Children and adults with LGS/DS were included from 25 EAP sites across the United States. Motor seizures were reduced by 50% and total seizures by 44%, supporting CBD as a long-term treatment option (Laux et al., 2019).

In their recent economic analysis, Elliot and coworkers (2020) compared the cost effectiveness of cannabinoid oil as an adjunctive treatment (added to clobazam and valproate), with adjunctive stiripentol or with clobazam and valproate alone, for the treatment of DS in children, concluding that adjunctive cannabinoid oil may be a cost-effective treatment for DS.

### Lennox-Gastaut Syndrome

In GWPCARE4 LGS double-blind placebo-controlled trials (Thiele et al., 2018 **)** patients were administered CBD at 20 mg/kg/day, while in GWPCARE3 trial to 10 or 20 mg/kg/day (Devinsky et al., 2018b) over a 14-week treatment period compared relative to the baseline period.

In GWPCARE3 study, 171 patients were randomized (86 to CBD and 85 to placebo). During the titration plus maintenance period, patients on CBD achieved a 44% median reduction in drop seizure frequency vs. 22% in the placebo group. In the same treatment period, patients had a 49% median reduction in non-drop seizures vs. 23% in the placebo group. Regarding the response for both seizure types (drop and non-drop), patients on CBD had a 41.2% median reduction in seizure frequency compared to 13.7% in the placebo group (Thiele et al., 2018).

In GWPCARE4, a total of 225 patients were randomized; 76 to 20 mg/kg/day, 73 to 10 mg/kg/day, and 76 to placebo. The reduction in seizure frequency was 41.9% and 37.2% in the 20 and 10 mg/kg/d CBD group, respectively, vs. 17.2% in the placebo group, revealing a significant difference in both CBD arms relative to placebo (GWPCARE4) (Devinsky et al., 2018c).

Based on the patient or caregiver Clinical Global Impression (CGI) scale, overall improvements were reported in patients of each trial: 58% patients (compared to 34% in the placebo group) in the study of Thiele et al. (2018), 57% and 66% in the 20 mg/kg/day and 10 mg/kg/day group, respectively (compared to 44% in the placebo group) in the study of Devinsky et al. (2018b) and 88% at 24 weeks (also similar at 38 and 48 weeks) in the open-label study of Savage et al. (2020).

### Tuberous Sclerosis Complex

Eighteen patients with a diagnosis of tuberous sclerosis complex (TSC) were enrolled in an expanded-access study of CBD. The median weekly seizure frequency decreased to 13.3 compared to 22.0 baseline observation period after 3 months of treatment with CBD. Considering total weekly seizure frequency, the median percent change was -48.8% (Hess et al., 2016).

In GWPCARE6 clinical trial, Epidiolex was used as add-on treatment in patients with TSC (Thiele et al., 2019). Patients were randomized to 20 mg/kg/day, 50 mg/kg/day, and placebo. Two-hundred one patients completed the study, and percent change in total seizure frequency decreased, respectively, by 48%, 48% and 27%. Responders (>50% seizure reduction) were 36, 40, and 22%. An overall improvement, based on the caregiver CGI scale, was reported for 69, 62, and 40% in the three groups, respectively. The lower dose of 20 mg/kg/day give a similar efficacy compared to the higher dose of 50 mg/kg/day dose but with less AEs, making the former preferable (Thiele et al., 2019).

### Infantile Spasms and Epileptic Spasms

Preliminary data, derived from a brief online survey, by Hussain et al. (2015), suggested that various formulations of CBD may show a potential efficacy through multiple resistant epilepsy syndromes, including infantile spasms (IS), but the authors concluded that the study “not represent compelling evidence of efficacy and safety” and because of the presence of limitations of paramount importance, with the suggestion of further controlled clinical trials.

Hussain et al. (2020), in a multicenter phase 2 study, enrolled 9 patients (median age, 23 months; range, 14–36 months) with resistant and long-standing IS (median duration, 17 months; range, 8–33 months), treated with synthetic pharmaceutical CBD. Eight of the nine patients had concomitant antiepileptic treatments upon entering the study, although none of them took clobazam. The results of efficacy demonstrated that only one patient had an immediate but temporary response, the other eight patients exhibited neither clinical nor electrographic response. The lack of a lasting response suggests that CBD is not highly effective in treating refractory IS. The Authors, despite the negative results in this small group of resistant IS, left the door open to new studies on younger patients with a shorter IS history.

Nevertheless, also in 2020, Herlopian et al. (2020) published an open-label study on CBD treatment of epileptic spasms (ES) in nine patients (average age, 9 years; range, 2–16 years) enrolled suffered from drug-resistant ES additionally to other types of seizures with an onset of ES at 4–21 months (average age, 8 months). Administration of CBD (10 to 50 mg/kg/day) in patients with ES corresponded to a positive clinical outcome in clinical and electrographic response with an adequate safety profile.

After six months of 18–84% reduction in seizures. Sixty-seven percent (6/9) of patients experienced a >95% reduction in seizure frequency in the first two weeks while at the end of the study, 67% (6/9) achieved >50% reduction in seizures frequency ES. CBD has also been effective in reducing the frequency of other types of seizures experienced by patients.

The seizure-free rate heightened from 33% at 2 months to 56% at 12 months. After nine months of treatment, only 22% experienced an increase in ES frequency after six months of 18-84% reduction in seizures. Sixty-seven percent (6/9) of subjects experienced a greater than 95% decrease in seizure frequency in the first two weeks while at the end of the study, 67% (6/9) had a greater than 50 reduction in seizures. CBD has also been effective in reducing the frequency of other types of seizures present in patients. Interestingly, eight of the nine (89%) patients had EEG studies prior to and after initiation of CBD. Three out of five patients (60%) had resolution in their hypsarrhythmia pattern.

In contrast to Hussain et al. (2020) results, pure CBD used in the last study (Herlopian et al., 2020) seems to be effective on clinical IS and EEG abnormalities. However, the small number of patient cohorts and the non-homogeneous clinical characteristics do not allow us to provide conclusive results on the different efficacy of pure CBD compared to synthetic CBD.

Despite 89% of the nine patients displaying adverse events such as drowsiness, diarrhea, ataxia, appetite loss, agitation, twitchiness, irritability, and elevated liver enzymes, none of the patients withdrew from the study.

### CDKL5 Deficiency and Other Developmental Epilepsies

Severe early onset epilepsies such as CDKL5 deficiency disorder (CDD) and other developmental epilepsies are extremely debilitating, largely due to the early-onset and refractory nature of the seizures. Evidence for cannabinoids is limited but growing, with multiple anecdotal reports and an open-label trial showing cannabidiol to be associated with a significant reduction in seizure activity.

#### CDKL5 Deficiency

Dale et al. (2019), in a recent review, reported that while research on severe refractory epilepsy syndromes confers a role for medicinal cannabis, specific research in patients with CDD is primarily represented by unverified anecdotal reports, therefore still limited.

Pamplona et al. (2018) performed a meta-analysis on the role of CBD in various drug-resistant pediatric epilepsy describing a significant improvement in seizure control including some with CDD, however, these studies (Devinsky et al., 2016; Szafarky et al., 2018) do not specify the effects on the subset of CDD patient as a single entity. To date, only one promising open-label study performed a quantitative analysis of the efficacy of CBD in children with severe drug-resistant epilepsies and onset in childhood, including CDD, as well as Aicardi, Dup15q, and Doose (Devinsky et al., 2018d). In particular, in CDD patients, the monthly average frequency of seizure decreased from 66 (n = 17) to 41% at week 12 (n = 11), and from 60 to 36% at week 48 (n = 10).

However, this study, although promising, needs further confirmation to formally evaluate the safety and efficacy of CBD in patients with CDD, in particular using larger placebo-controlled randomized trials (Devinsky et al., 2018d).

#### Doose Syndrome

In a study by Porter and Jacobson (2013) the parents of 4 patients with Doose Syndrome reported clinical improvement in 3 patients with more than 80% decrease of seizures (in two of these complete seizure freedom) after a follow-up di 2-4 months while 1 patient was unresponsive after 2 weeks of CBD. Press et al. (2015), in 75 patients with drug-resistant epilepsies reported that all three patients with Doose Syndrome were unresponsive to CBD. Nevertheless, albeit considering the small number, Devinsky et al. (2018d) based on an open-label trial of a drug-resistant form of epilepsy in which seven patients had Doose syndrome had promising results. These patients presented a reduction of seizure frequency passing from a median convulsive seizure frequency pre-CBD of 60.8 and a total seizure frequency of 64.7 to a median reduction of convulsive seizures from baseline of 58.6% by week 12 and 28.8% by week 48 after CBD.

#### Dup15q Syndrome

15q duplication syndrome and related disorders (dup15q) are caused by at least one extra maternally derived copy of the Prader-Willi/Angelman critical region (PWACR) within chromosome 15q11.2-q13.1. Clinically, Dup15q is characterized by hypotonia, motor delays, intellectual disability, autism spectrum disorder (ASD), and epilepsy including drug-resistant form (Finucane et al., 1993).

Devinsky et al. (2018d) based on an open-label trial of a drug-resistant form of epilepsy in which eight patients with had Dup15q variant a reported median convulsive seizure baseline frequency of 118.5 (n = 8, IQR: 32–231) and a total seizure frequency of 149.1 (n = 8, IQR: 57–313). In the Dup15q subgroup, the median number of seizures decreased from baseline (118.5 [n = 8], IQR: 18–241) to week 12 (48.8 [n = 7], IQR: 5–99), with no change from week 12 to week 48 (53.02 [n = 6], IQR: 7–207) (χ2(2) = 3.00, p = 0.223). Those with the Dup15qmutation have a reported median convulsive seizure decrease from baseline [n = 8] of 25% by week 12 (n = 7; IQR: -10–71) and 38.4% by week 48 (n = 6, IQR: -13–88). The Dup15q subgroup had a 38% responder rate, which persisted through week 48.

#### Sturge-Weber Syndrome

Sturge-Weber syndrome is characterized by leptomeningeal vascular malformations, refractory epilepsy, stroke (s) and cognitive disabilities. In preclinical models, CBD has been shown to have a possible anticonvulsant, antioxidant and neuroprotective action (Kaplan et al., 2017).

Kaplan et al. (2017) suggested that CBD may be well tolerated and provides initial data as an adjunctive medication for resistant epilepsy of Sturge-Weber syndrome. Three out of five subjects reported mild side effects considered related to CBD. Three out of five patients, demonstrating a better CBD response had bilateral brain involvement, were treated with two or more anticonvulsants and low-dose aspirin at the entry and had significant cognitive, neurological, behavioral or mood issues; the remaining two patients were removed from the study for lack of efficacy.

#### Migrating Focal Seizures Associated With KCNT1 Mutations

Epilepsy of Infancy with Migrating Focal Seizures (EIMFS) is a rare, developmental and epileptic encephalopathy most commonly associated with mutations in KCNT1, a potassium channel (Coppola, 2013; Auvin et al., 2016). Saade and Joshi (2015) described the beneficial effect of CBD in sustained seizure reduction with the addition of CBD to the antiepileptic regimen in an infant with EIMFS (tested only for mutations in the SCN1A gene, while not for KCNT1).

Recently, Poisson et al. (2020) evaluated CBD response in three patients with EIMFS secondary to KCNT1 mutations; two subjects showed no benefit and voluntarily discontinued CBD. One patient showed an overall decrease in seizure frequency, however, had significant decrease in seizure intensity with the possible progression of development.

## Adverse Effects of Cannabis Extract and CBD

The most frequent side effects reported using Cannabis extract are sleepiness, fatigue, nausea diarrhea ad decrease appetite. There are concerns about exposition to THC and its effect on brain development and long-term data moreover indicate a possible negative effect on cognitive and behavioral performance (Lagae, 2020). However, no conclusive data can be derived from available studies, given the methodological limitation, the unknown dosage of THC in artisanal products, the different duration of exposure, genetic factor, the combined use of other antiepileptic drugs, and the seizure control.

Considering pure CBD, 86% of patients in CBD groups versus 76% in placebo groups reported AEs in RCTs. However, the vast majority of AEs were mild and most of them appeared within the first two weeks of treatment.

The most frequent are somnolence, decreased appetite, pyrexia, and diarrhea, followed by other less frequent AEs such as vomiting, fatigue, and upper respiratory infections.

Serious AEs, such as somnolence, pyrexia, convulsion, rash, lethargy, and elevated transaminases (>3 times), were far less common, affecting 19% of CBD groups and 9% of placebo groups.

Elevated transaminases occurred in 16% of patients in the CBD groups and 1% in the placebo groups. The majority of the cases with elevated transaminases were patients concomitantly taking valproate. No seizure worsening, suicidal ideation, or deaths related to the treatment were reported (Devinsky et al., 2017a; Thiele et al., 2018; Devinsky et al., 2018c).

The long-term AEs are currently unknown.

In the TSC trial with the higher dose of 50 mg/kg/day CBD (Thiele et al., 2019), the most common AEs were diarrhea, decreased appetite, and somnolence, and treatment discontinuation due to AEs occurred in 11, 14, and 3%, respectively. Elevated liver enzymes were reported in 12% (n = 9) and 25% (n = 18) in the 25 and 50 mg/kg/day, respectively (of those, 81% were also taking valproate).

## Directions for Use and Future Research

Drug-resistant epilepsies in children represent a challenge, both for efficacy and safety aspects. In the landscape of the pediatric drug-resistant epilepsy responsive to CBD treatment, few conditions, such as Dravet and Lennox-Gastaut syndromes, have given good scientific evidence showing a good therapeutic response. Nevertheless, in this paper, we summarized the current state of evidence and indications for CBD therapy in the most frequent epileptic syndromes in childhood, not just in Dravet and Lennox-Gastaut syndromes.

Support for CBD use in pediatric epilepsies should take into account the CBD mechanisms of action and the knowledge of the epileptogenic mechanisms in the single specific epileptic syndrome, and increasing into the knowledge of the pharmacogenomic profile of CBD-AEDs interactions, starting from animal models studies.

Personalized medicine, through the study of pharmacogenomics, could provide useful information for the therapeutic choice and recognition of the patients (and specific epileptic syndromes) most responsive to CBD therapy; also to provide information on the best association between CBD and other AEDs in the specific individual affected by epilepsy.

In the case of weak pathophysiological hypotheses, the clinical studies should identify a specific subpopulation, affected by specific epileptic syndromes, which may benefit from the CBD treatment.

Increase in genetic knowledge that underlies and regulates the pharmacodynamics and pharmacokinetics of CBD and drug-drug interaction will significantly improve the choice of the therapeutic CBD prescriptions, both as monotherapy and polytherapy, in children with epilepsy.

## Conclusions

CBD has been used as an anticonvulsant for at least 4000 years (Russo, 2017). Its use for medicinal purposes is now authorized in many different countries around the world. THC is a controlled substance and according to EU law, CBD products must not contain more than 0.2% THC (Arzimanoglou et al., 2020). In Italy from November 2015, cannabis may be prescribed with medical prescription. Italian legislation has approved regulations regarding the administration of medical Cannabis for specific medical conditions (pain therapy, chemotherapy/radiotherapy-induced nausea and vomiting, cachexia, anorexia, cancer patients, AIDS, glaucoma, and Tourette’s syndrome (Baratta et al., 2019).

There are concerns about exposure to THC and its effect on brain development. Frequent side effects reported were sleepiness, fatigue, nausea diarrhea and decreased appetite, somnolence, pyrexia, and diarrhea, followed by other less frequent events such as fatigue, upper respiratory infections, convulsion, rash, lethargy, and elevated transaminases (>3 times); developmental regression abnormal movements and status epilepticus have also been described. Long-term data indicate a possible negative effect on cognitive and behavioral performance (Lagae, 2020). Unfortunately, appropriate pediatric dose and pharmacokinetics continue to make the authorization of cannabis-based therapies to children a challenge (Huntsman et al., 2020).

Nonetheless, the role that the endocannabinoid system plays in epileptogenesis, encourages to investigate the use of exogenously cannabinoids to treat epileptic children (Cheung et al., 2019; Huntsman et al., 2020).

CBD, both in isolation as a pharmaceutical-grade preparation or as part of a CBD-enriched cannabis herbal extract, shows beneficial effects in decreasing seizure frequency in children with drug-resistant epilepsy. Recently, in patients with Lennox-Gastaut syndrome and Dravet syndrome (Devinsky et al., 2016; Devinsky et al., 2017a; Devinsky et al., 2017b; Rosenberg et al., 2017b; Thiele et al., 2018; Stockings et al., 2018; Lattanzi et al., 2019; Lattanzi S. et al., 2020) have been conducted a placebo-controlled RCTs with a pure form of CBD, which gave good results (Class I evidence). Later, CBD was approved by the FDA as an add-on antiepileptic drug in 2 years old children with Dravet syndrome and Lennox-Gastaut syndrome. Subsequently, it was approved also by the EMA in 2019. The purified preparation of CBD is available from GW Pharmaceuticals plc, named Epidiolex/Epidyolex. It has been shown to have positive effects against a wide spectrum of seizures from experimental studies (Rosenberg et al., 2017a).

Although to date just preliminary results and weak scientific evidence are available for many other epileptic conditions, we reported above also every specific pediatric epileptic condition in which CDB was be tried, with more or less encouraging data. CBD investigations in pediatric age, better evaluation of the incidence and the prevalence of epileptic syndromes age-related, together with increased knowledge of their natural course, and the development of new end points could provide some suggestions for future improvements for the therapeutic utilization of CBD therapy in epileptic children.

## Author Contributions 

UR and PaP directed the review and were responsible for the overall guidance. All authors contributed to the article and approved the submitted version. The article’s content has been made by consensus among all the authors.

## Conflict of Interest

NS received grant support from GW Pharma, Biomarin, Livanova, Xenon, and Marinus and a speakers fee from GW Pharma, Livanova, Biomarin, and Zogenix.

The remaining authors declare that the a speakers was conducted in the absence of any commercial or financial relationships that could be a potential conflict of interest.
